# Association Between Gamma-Glutamyl Transferase and Mild Cognitive Impairment in Chinese Women

**DOI:** 10.3389/fnagi.2021.630409

**Published:** 2021-02-10

**Authors:** Zhaoyang Tang, Xueyu Chen, Wenran Zhang, Xiangfu Sun, Qingzhi Hou, Yuejin Li, Xia Feng, Yanru Chen, Jian Lv, Long Ji, Guoyong Ding, Dong Li

**Affiliations:** ^1^Department of Epidemiology, School of Public Health, Shandong First Medical University & Shandong Academy of Medical Sciences, Taian, China; ^2^Taian Traffic Hospital, Taian, China; ^3^The Second Affiliated Hospital of Shandong First Medical University, Taian, China

**Keywords:** mild cognitive impairment, female population, diagnostic marker, cross-sectional study, gamma-glutamyl transferase

## Abstract

**Background:** Dementia, as a global public health problem, is becoming increasingly serious. As a precursor of dementia, mild cognitive impairment (MCI) plays an important role in the diagnosis and prevention of dementia. Recent studies have found a correlation between gamma-glutamyl transferase (GGT) levels and cognitive function in men. The relationship between GGT levels and cognitive function in women remains unclear because GGT activity and expression differ between the sexes.

**Method:** We recruited a total of 2,943 Chinese women from Jidong and Taian in 2019. We grouped the participants according to GGT levels, diagnosed MCI using the Montreal Cognitive Assessment (MOCA) scale, and modeled the study outcomes using logistic regression to explore the relationship between GGT level and MCI. We also analyzed the interaction of obesity, sleep duration, and hyperuricemia with GGT in the development of MCI.

**Results:** The prevalence of MCI increased with increasing GGT level, from the lowest quartile to the highest quartile of GGT: 8.4% (66/786), 14.2% (119/840), 17.6% (108/613), and 21.4% (151/704), respectively. At the same time, as GGT levels increased, so did the risk of MCI. In the fully adjusted model, compared with those for participants in the lowest GGT quartiles, the odds ratios (ORs), and 95% confidence intervals (CIs) for MCI for participants in the second, third, and fourth GGT quartiles were 1.49 (1.04–2.12), 1.53(1.06–2.21), and 1.88 (1.33–2.65), respectively. The risk of developing MCI was further increased in people with high GGT levels who were obese (OR = 1.96, 95% CI: 1.39–2.76, *P* < 0.001), slept less (OR = 1.91, 95% CI: 1.35–2.71, *P* < 0.001), had high levels of uric acid (OR = 1.55, 95% CI: 1.03–2.32, *P* < 0.001), or after menopause (OR = 2.92, 95% CI: 2.07–4.12, *P* < 0.001).

**Conclusion:** We found that MCI is more common in women with elevated GGT levels, so GGT could be a potential diagnostic marker for MCI. Meanwhile, our findings indicated that women with high GGT levels had an increased risk of MCI when they were obese, sleep deprived, had high serum uric acid (SUA) levels or underwent menopause.

## Introduction

With the aging of the population, dementia has become a major global public health problem and caused a huge disease burden (WHO, 2017). The global prevalence of dementia is expected to rise from 30 million in 2010 to 106 million in 2050 (Brookmeyer et al., 2007). Mild cognitive impairment (MCI) refers to the clinical condition between normal aging and dementia in which persons experience memory loss to a greater extent than one would expect for their age, yet they do not meet currently accepted criteria for clinically probable dementia (Petersen, 2001). Therefore, MCI is regarded as the prodromal stage of dementia; when people have MCI, their rate of conversion to dementia will be considerably accelerated compared with that of healthy age-matched individuals (Petersen, 2000; Thompson and Hodges, 2002; Ding et al., 2015).

Gamma-glutamyl transferase (GGT) is routinely used in clinical practice as an indicator of potential hepatic or biliary diseases, as it is mainly found in the liver and plays an important role in maintaining the intracellular concentration of glutathione (Kristenson et al., 1985; Forman et al., 2009; Kunutsor, 2016). Recently, the serum level of GGT has been reported to be associated with vascular diseases, which are considered to have pro-oxidant and proinflammatory properties (Emdin et al., 2005; Kunutsor et al., 2014, 2015; Jeon et al., 2020). However, inflammation and oxidative stress are the most likely pathways leading to cognitive impairment (Zafrilla et al., 2006; Gackowski et al., 2008). Therefore, GGT levels might be associated with an increased risk of dementia. A previous cohort study showed that serum GGT was log-linearly associated with the risk of dementia, and the risk of dementia increases with increasing GGT level in the male population (Kunutsor and Laukkanen, 2016). Another longitudinal study of older adults also showed that increased GGT levels are associated with cognitive decline at the end of life (Praetorius Bjork and Johansson, 2018). However, the result of a population-wide genetic study could not confirm a causal effect of GGT on the risk of dementia (Kunutsor et al., 2018). Therefore, the relationship between GGT and cognitive impairment is far from clear, especially among female individuals, among whom there is a lack of related research.

A systematic review of 75 studies in 2013 showed that the prevalence of dementia was significantly higher in women, with a prevalence rate that was 1.65 times higher among women than men (Chan et al., 2013); as a result, women are more likely to suffer from cognitive impairment. At the same time, an ovariectomized mouse model found that GGT activity might be related to estrogen (Zarida et al., 1994), which suggests that women not only face a higher risk of dementia but also are more sensitive to GGT activity.

In summary, owing to the sex-specific expression of GGT and the unknown relationship between GGT and MCI (Schiele et al., 1977), we collected data on GGT levels, cognitive function and general condition information from 2,943 women from two different Chinese cities to explore the association between GGT and MCI in the female population.

## Methods

### Study Participants

**Figure 1:**
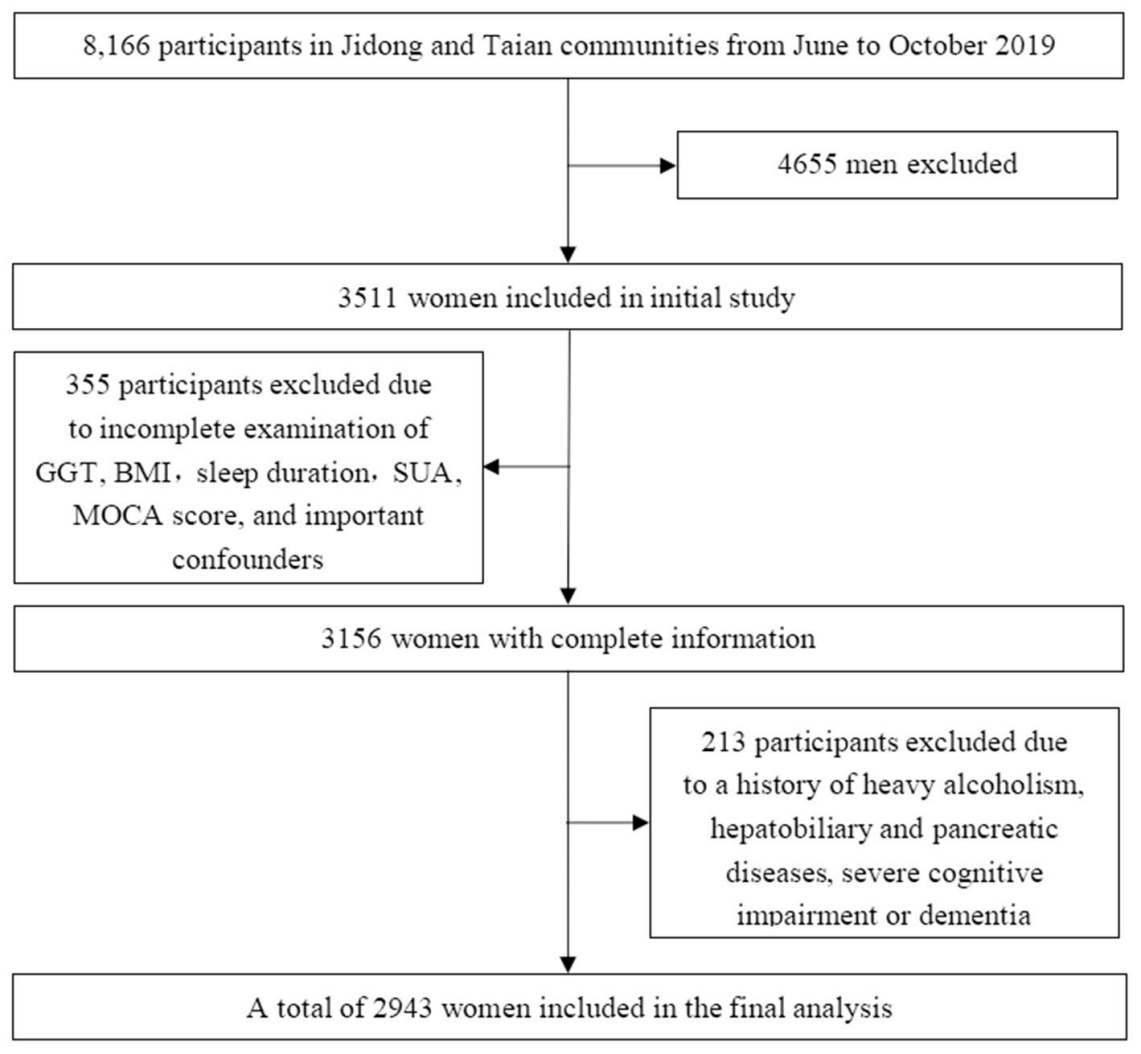
Flow chart of the study.

In 2019, a total of 8,166 participants from Jidong (*n* = 6,154) and Taian (*n* = 2,012), including 4,655 male participants and 3,511 female participants, underwent health checks. Among the 3,511 female participants, individuals with missing data on GGT, body mass index (BMI), sleep duration, serum uric acid (SUA), Montreal Cognitive Assessment (MOCA) score, or important confounders (*n* = 355, 10.1%); heavy alcoholism (defined as 30 g/day, *n* = 25, 0.7%); hepatobiliary and pancreatic diseases including cancer and viral hepatitis (*n* = 119, 3.3%); and a history of dementia (*n* = 6, 0.2%) were excluded from this study. In our study, participants with severe cognitive impairment (defined as a chief complaint of memory impairment and a MOCA score <18, *n* = 66, 1.9%) were excluded, even if they were not diagnosed with dementia. Eventually, a total of 2,943 female participants were included in our study (Figure 1).

The study was conducted in accordance with the guiding principles of the Helsinki Declaration and approved by the Ethics Committee of Jidong Oilfield Inc. Medical Centers and Taian Traffic Hospital. Written informed consent was obtained from all participants.

### Assessment of Mild Cognitive Impairment

Previous studies have found that the MOCA scale is more sensitive in MCI screening than other scales, and the specificity is not significantly different from that of other scales (Dong et al., 2010, 2012; Kasten et al., 2010). Therefore, we decided to use the MOCA scale to evaluate MCI. In this study, using the Chinese version of the MOCA scale and cutoffs for the Chinese population. The MOCA scale is a rapid tool for detecting MCI, which is assessed from different cognitive domains, they include attention, memory, executive function, language, visuospatial ability, abstract thinking, numeracy, and orientation. Each MOCA is limited to 10 min, with a maximum score of 30. We defined no subjective cognitive decline and a MOCA score ≥26 as normal (Dong et al., 2010, 2012; Kasten et al., 2010), and complaints of memory loss and a MOCA score of more than 18 and <26 points as MCI (Nasreddine et al., 2005). Considering the effect of education on cognitive function, those with 12 years or less of education had 1 point added to their scores. The MOCA scale was administered by specially trained investigators.

### Assessment of Gamma-Glutamyl Transferase (GGT) and Serum Uric Acid (SUA)

Blood samples were collected by venipuncture from the large antecubital veins in the morning after overnight fasting. All blood samples were stored in vacuum tubes containing ethylenediaminetetraacetic acid (EDTA). GGT and SUA levels were determined using an autoanalyzer (Hitachi 747; Hitachi, Tokyo, Japan) with the kinetic and uricase-peroxidase methods, respectively, in the laboratories of Jidong Oilfield Hospital and Taian Traffic Hospital.

### Assessment of Covariates

A series of standardized questionnaires, clinical examinations, and laboratory tests were used to collect basic information (Zhang et al., 2013). A standardized questionnaire was administered by well-trained interviewers to collect subjects' information. Demographic variables, including age, sex, education level and histories of hypertension, diabetes mellitus, dyslipidemia and hepatobiliary, and pancreatic diseases, were collected through the questionnaire. We also asked them about their menstrual history to see if they had gone through menopause and their age at menopause. According to self-reported information, alcohol consumption was classified as heavy drinker (alcohol consumption ≥30 g/day), mild-moderate drinker (alcohol consumption <30 g/day) or abstainer (alcohol consumption = 0 g/day) (Yoo et al., 2020), and smoking status was classified as non-smoker or having quit more than 1 year ago or current smoker or having quit <1 year ago. BMI was defined based on the measured height and weight and calculated as weight (kg)/height (m^2^). Education was categorized into illiterate or primary school, middle school, or university or above. The Pittsburgh Sleep Quality Index Questionnaire was used to measure sleep quality and duration (Mollayeva et al., 2016). Sleep duration was classified as <6, 6–7, 7–8, and >8 h, and adequate sleep or insufficient sleep were defined based on whether the sleep duration was greater than the median of 7 h (Papandreou et al., 2019). Hypertension was defined as the use of antihypertensive drugs, a self-reported history, diastolic blood pressure ≥90 mmHg, or systolic blood pressure ≥140 mmHg. Diabetes mellitus was defined as current treatment with insulin or an oral hypoglycemic agent, presence of a history of diabetes, or fasting blood glucose level ≥7.0 mmol/L (126 mg/dL). Dyslipidemia was defined as current use of lipid-lowering therapy, a self-reported history, or serum levels of triglyceride (TG) ≥1.7 mmol/L, total cholesterol (TC) ≥5.18 mmol/L, high-density lipoprotein (HDL) <1.04 mmol/L, or low-density lipoprotein (LDL) ≥3.37 mmol/L (Lv et al., 2016).

### Statistical Analysis

The Kolmogorov-Smirnov test was used to measure the normality of the distribution of the test variables. Continuous variables with normal distributions were expressed as the mean ± standard deviation (SD). Continuous variables that did not have a normal distribution were represented by the mean and interquartile range. Categorical variables were presented as frequencies and percentages. We compared the baseline characteristics of different serum GGT quartile groups using one-way ANOVA or the Kruskal-Wallis rank-sum test for normally or non-normally distributed continuous variables, respectively, and the chi-square test or Fisher's exact test for categorical variables. We also compared the baseline characteristics of groups with and without MCI using Student's *t*-test or the Mann-Whitney *U*-test for normally or non-normally distributed continuous variables, respectively, and the chi-square test or Fisher's exact test for categorical variables. Logistic regression model was used to evaluate the association between risk factors (GGT, BMI, sleep duration, SUA, and estrogen level) and MCI by calculating odds ratios (ORs) with 95% confidence intervals (CIs). The 25th, 50th, and 75th percentiles of GGT nodes were selected by using the restricted cubic spline method, and the confounding factors were adjusted by using the 25th percentile (12 U/L) as the reference. A dose-response diagram was also drawn by the GGT levels as horizontal coordinates, and the corresponding OR values as vertical coordinates. Nomogram was based on the results of logistic regression analysis. We used the postestimation Wald test in the multivariable-adjusted logistic model to obtain an omnibus *P*-value for interaction, the models were adjusted for age, education, smoking status, alcohol consumption, hypertension, hyperlipidemia, and diabetes mellitus.

The statistical analyses were performed using SAS software, version 9.4 (SAS Institute Inc., Cary, NC, USA), and the significance level was set as *P* < 0.05.

## Results

### Baseline Characteristics of the Study Participants

A total of 2,943 participants from Jidong (2,821) and Taian (122) eventually enrolled in our study (Figure 1). We found no statistically significant differences between the populations from the two centers in terms of GGT, sleep duration, BMI, smoking status, education level, hypertension, diabetes mellitus, dyslipidemia, SUA, total cholesterol, triglycerides, HDL-C, LDL-C, and estrogen level (*P* > 0.05). Although there was a significant difference in the age of potential subjects from the two centers, there was no significant difference in the ages of the MCI patients from the two centers (Supplementary Table 1). Therefore, we combined the patients from the two centers for the analysis.

We compared baseline characteristics by different quartiles of serum GGT activity (Table 1). There were 14 factors that were significantly associated with serum GGT levels, including age, sleep duration, BMI, smoking status, education level, hypertension, diabetes mellitus, dyslipidemia, SUA, total cholesterol, triglycerides, HDL-C, LDL-C, and estrogen level (*P* < 0.05). We considered all of these possible confounding variables when evaluating the independent relationship between GGT and MCI.

**Table 1:** Comparison of baseline characteristics according to quartiles of serum GGT.

**Characteristic**	**GGT level**					
	**Q1 (*n =* 786)**	**Q2 (*n =* 840)**	**Q3 (*n =* 613)**	**Q4 (*n =* 704)**	***P***	***P* for trend**
GGT(U/L)	10.6 ± 1.3	14.4 ± 1.1	19.1 ± 1.7	42.6 ± 3.3	<0.001	<0.001
Age (years)	42.1 ± 12.2	45.5 ± 13.1	48.5 ± 13.5	50.1 ± 12.8	<0.001	<0.001
Education level (*n*, %)					<0.001	<0.001
<6 years	33 (4.2)	47 (5.6)	49 (8.0)	49 (7.0)		
6–12 years	196 (24.9)	273 (32.5)	238 (38.8)	339 (48.2)		
>12 years	557 (70.9)	520 (61.9)	326 (53.2)	316 (44.9)		
Sleep duration (*n*, %)					<0.001	<0.001
<7 h	281 (35.8)	356 (42.4)	296 (48.3)	329 (46.7)		
≥7 h	505 (64.2)	484 (57.6)	317 (51.7)	375 (53.3)		
BMI (*n*, %)					<0.001	<0.001
<25 kg/m^2^	111 (14.1)	205 (24.4)	221 (36.1)	308 (43.8)		
≥25 kg/m^2^	675 (85.9)	635 (75.6)	392 (63.9)	396 (56.3)		
Current smoker (*n*, %)	14 (1.8)	44 (5.2)	32 (5.2)	54 (7.7)	<0.001	<0.001
Mild-moderate drinking (*n*, %)	5 (0.6)	8 (1.0)	5 (0.8)	10 (1.4)	0.459	0.162
Hypertension (*n*, %)	32 (4.1)	58 (6.9)	93 (15.2)	126 (17.9)	<0.001	<0.001
Dyslipidemia (*n*, %)	13 (1.7)	31 (3.7)	50 (8.2)	78 (11.1)	<0.001	<0.001
Diabetes (*n*, %)	13 (1.7)	21 (2.5)	32 (5.2)	56 (8.0)	<0.001	<0.001
UA(umol/L)	271.4 ± 55.6	291.9± 65.2	305.1 ± 66.9	328.0 ± 76.6	<0.001	<0.001
TC (mmol/L)	4.7 ± 1.6	5.7 ± 6.8	5.6 ± 5.9	6.7± 10.7	<0.001	<0.001
Triglycerides (mmol/L)	1.1 ± 0.5	1.3 ± 0.7	1.6 ± 1.0	2.1 ± 1.7	<0.001	<0.001
HDL-C (mmol/L)	1.3 ± 0.2	1.3 ± 0.3	1.2 ± 0.3	1.2 ± 0.2	<0.001	<0.001
LDL-C (mmol/L)	1.8 ± 0.7	1.9 ± 0.7	2.1 ± 0.8	2.3 ± 0.8	<0.001	<0.001
Menopause (*n*, %)	168 (21.4)	268 (31.9)	254 (41.4)	302 (42.9)	<0.001	<0.001
MCI (*n*, %)	66 (8.4)	119 (14.2)	108 (17.6)	151 (21.4)	<0.001	<0.001

*Q1, quartile 1 (n = 786): ≤ 12 U/L; Q2, quartile 2 (n = 840): 13-16 U/L; Q3, quartile 3 (n = 613): 17-22 U/L; Q4, quartile 4 (n = 704): >22 U/L*.

At the same time, baseline characteristics were compared in women participants with and without MCI. Significant differences were found in age, sleep duration, BMI, smoking status, education level, hypertension, diabetes mellitus, dyslipidemia, triglycerides, SUA, LDL-C, GGT, and estrogen level (*P* < 0.05). Supplementary Table 2 clearly shows the association between baseline characteristics and MCI.

### Association Between GGT and MCI in Women

The prevalence of MCI in the participants increased with increasing GGT level, being 8.4, 14.2, 17.6, and 21.4% in the lowest to highest quartiles of GGT, respectively (Table 1).

**Figure 2:**
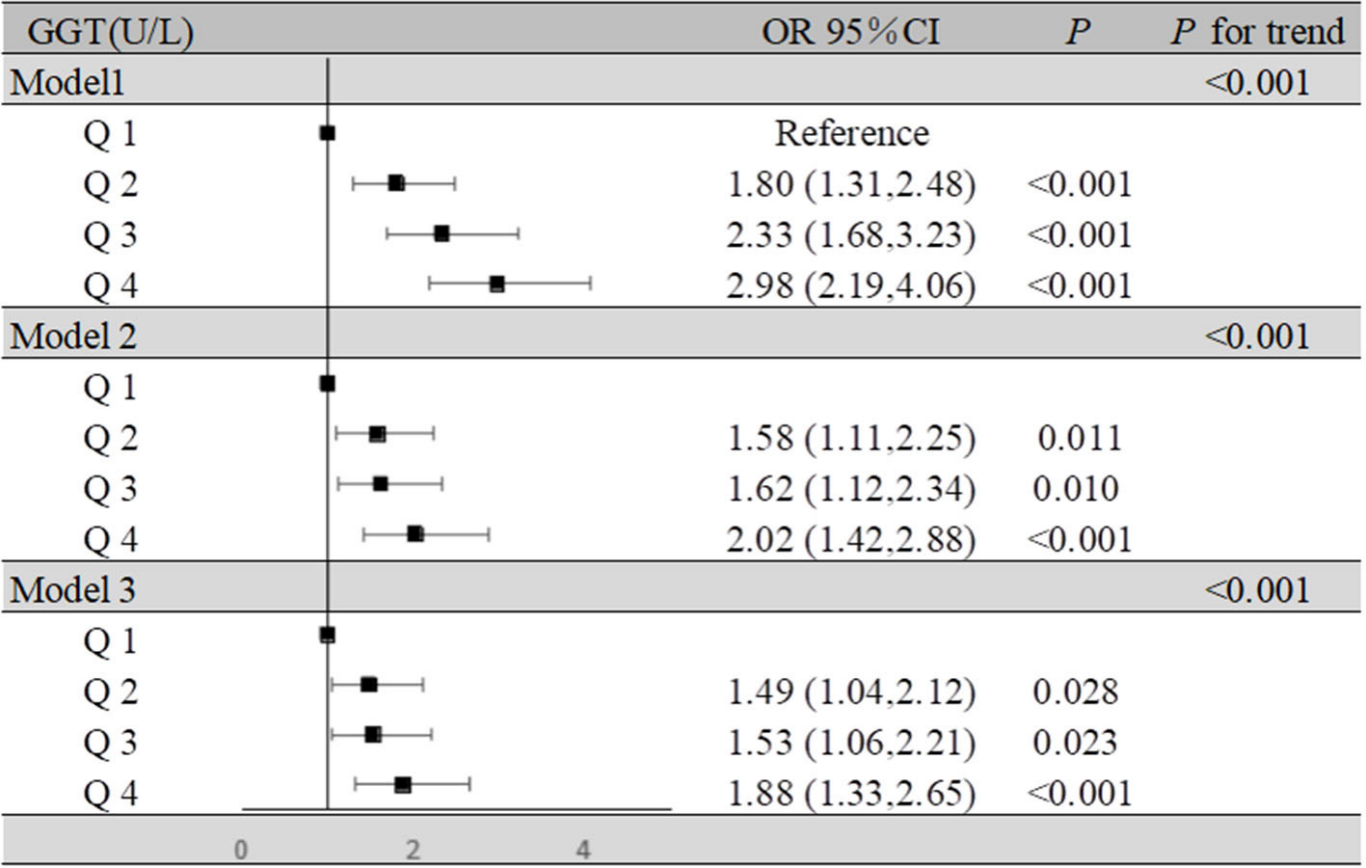
Association of GGT levels and MCI in the participants. Model 1: unadjusted. Model 2: adjusted for age, BMI, education, sleep duration, smoking status, and alcohol consumption. Model 3: adjusted for age, BMI, education, sleep duration, smoking status, alcohol consumption, hypertension, hyperlipidemia, diabetes mellitus, serum uric acid, and estrogen level.

**Figure 3:**
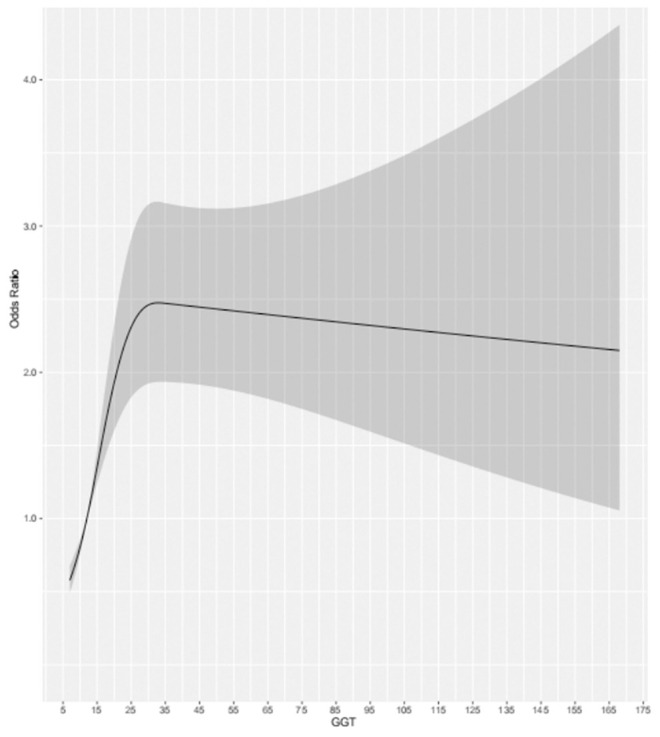
Dose-response relationship between the risk of MCI and changes in GGT level.

For the participants in the present study, compared to the lowest quartile, the highest quartile of GGT exhibited a positive association with MCI risk (OR = 1.88, 95% CI: 1.33–2.65, *P* < 0.001) in the fully adjusted model. At the same time, we found that as GGT levels increased, the ORs and 95% CIs for MCI of the participants in the second, third, and fourth GGT quartiles increased, respectively, compared with those of the participants in the lowest GGT quartile. Moreover, in all three models of the participants, the *P* for trend across quartiles was <0.001 (Figure 2). The dose-response relationship between GGT level and risk of MCI was analyzed by using the restricted cubic spline regression model. And the risk of MCI increased with increasing GGT level when the GGT level was below 35 μ/l, and when the GGT level was higher than 35 U/l, the risk of MCI remained high but decreased slightly with increasing GGT level (Figure 3).

### Effect of BMI, Sleep Duration, Serum Uric Acid, and Estrogen Level on the Relationship Between GGT and the Risk of MCI

In our study, obesity, insufficient sleep, hyperuricemia, and menopause all increased the risk of MCI of the participants. The OR and 95% CI for MCI due to obesity was 1.32 (1.04–1.69) in the fully adjusted model. Likewise, insufficient sleep also increased the risk of MCI, and the OR and 95% CI for MCI due to insufficient sleep was 1.33 (1.05–1.69) in the fully adjusted model. For hyperuricemia, the OR and 95% CI for MCI was 1.34 (1.01–1.77) in the fully adjusted model. Reduced estrogen levels also affect the development of MCI in postmenopausal women, the OR and 95% CI for MCI due to menopause was 2.28 (1.78–2.91) in the fully adjusted model (Supplementary Table 3). We also found that obesity, insufficient sleep, hyperuricemia, and menopause were significant risk factors for MCI according to the nomogram based on the logistic regression results for all factors (Supplementary Figure 1).

**Figure 4:**
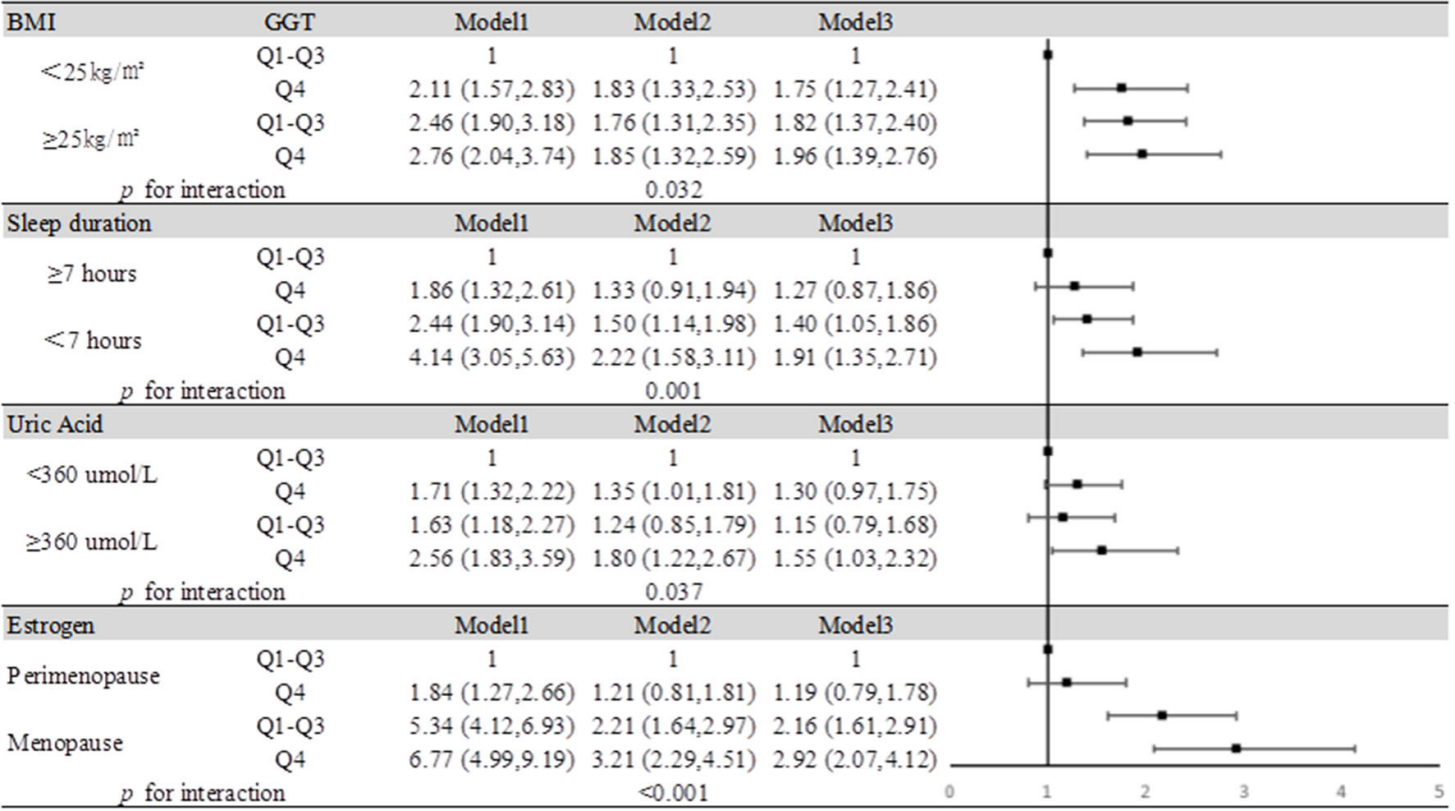
Impact of serum GGT level on MCI according to BMI, sleep duration, uric acid, and estrogen. Model 1: unadjusted. Model 2: adjusted for age, education, smoking status, and alcohol consumption. Model 3: adjusted for age, education, smoking status, alcohol consumption, hypertension, hyperlipidemia, and diabetes mellitus.

We summarized the results of the analysis on the effects of the interactions between serum GGT and the presence of obesity, insufficient sleep, and hyperuricemia on the risk of MCI in Figure 4. Through interaction analysis, we found that women with high GGT levels had an increased risk of MCI when they were obese (OR = 1.96 for obese women with Q4 GGT compared to nonobese women in Q1-Q3 GGT, *P* for interaction = 0.032), lacked sleep (OR = 1.91 for women who slept less and had Q4 GGT compared to women who slept more and had Q1-Q3 GGT, *P* for interaction = 0.001), had a high level of SUA (OR = 1.55 for women with high UA levels and Q4 GGT compared to women with low UA levels and Q1-Q3 GGT, *P* for interaction = 0.037) or after menopause (OR = 2.92 for postmenopausal women and Q4 GGT compared to premenopausal women and Q1-Q3 GGT, *P* for interaction <0.001). Considering women with high GGT levels, when they were obese, lack of sleep, had high levels of uric acid or after menopause, we obtained higher OR values than that we did for women with high GGT levels alone (OR = 1.19, 95% CI: 1.07–1.33). So obesity, lack of sleep, hyperuricemia, and menopause all had subadditive effects on the increased risk of MCI associated with GGT (Supplementary Table 3, Figure 4).

## Discussion

In our study, we found that GGT might be an independent risk factor for MCI in the female population, and as GGT levels increased, so did the risk of MCI. At the same time, we also found that women with high GGT levels were at increased risk of developing MCI when they were obese, lacked sleep, had high levels of uric acid or after menopause. BMI, sleep duration and uric acid may interact with GGT in the development of MCI.

Our study found an association between higher GGT and higher risk of cognitive impairment in the female population, which is consistent with previous studies in the male population (Kunutsor and Laukkanen, 2016). A study of GGT and Parkinson's disease is also consistent with our finding, that GGT may contribute to neurodegenerative disease in the female population (Yoo et al., 2020). However, contrary to our findings, a genetic study found no clear link between GGT and cognitive function (Kunutsor et al., 2018), possibly because that study did not include a subgroup analysis based on sex, which is essential because of the finding from previous study that estrogen may affect GGT activity in the body (Zarida et al., 1994).

The potential proinflammatory and pro-oxidative effects of GGT might be an important cause of the increased risk of MCI related to GGT (Kunutsor et al., 2014, 2015). Additionally, GGT levels were directly involved in atheromatous plaque formation, which has also been implicated as an underlying link in the pathogenesis of cognitive impairment (Breteler, 2000; Paolicchi et al., 2004; Franzini et al., 2009).

Through the analysis of the baseline data, we found that the differences in age, education, sleep duration, BMI, hypertension, dyslipidemia, diabetes, SUA, triglycerides. LDL-C, and estrogen decline were statistically significant not only in the MCI and non-MCI groups (Supplementary Table 2), but also in the different GGT level groups (Table 1). However, previous studies found that only BMI (Carter et al., 2019; Cho et al., 2019), sleep (D'Rozario et al., 2020; Zhang et al., 2020), uric acid (Zhang et al., 2015; Ya et al., 2017; Tana et al., 2018) and estrogen level (D'Rozario et al., 2020; Zhang et al., 2020) affected both GGT and MCI. Moreover, after we used logistic regression to establish the nomogram, we found that obesity, lack of sleep, high uric acid levels, and menopause were risk factors for MCI. Therefore, the effects of the interaction of obesity, lack of sleep, high uric acid levels, and menopause with GGT on MCI was analyzed by demographic, lifestyle, and biochemical parameters. Interestingly, when we constructed nomograms, we found that mild-moderate alcohol consumption may have a weak protective effect on MCI, consistent with previous research on the relationship between alcohol consumption and Alzheimer's disease (AD) (Venkataraman et al., 2016).

Direct release in the context of hepatocyte injury is not the sole cause of serum GGT elevation. GGT elevation may also be due to factors such as obesity related to serum GGT activities that occur and lead to increased expression or decreased breakdown. A previous study showed that the level of GGT expression in obese people may be different from that in normal people (Carter et al., 2019). Moreover, obesity also plays an important role in neurodegenerative diseases by inducing insulin resistance (Pugazhenthi et al., 2017). These findings make it possible for BMI and GGT to play a synergistic role in the development of MCI.

Sleep may cause changes in GGT levels because of its interaction with obesity, and a recent nematode model study demonstrated a synergistic effect between insomnia and obesity (Grubbs et al., 2020; Zhang et al., 2020). Therefore, lack of sleep may also lead to GGT elevation. In the same way, lack of sleep is a major cause of cognitive impairment. In summary, the mechanism of sleep deprivation affecting MCI may be partially enhanced by GGT.

The levels of serum UA, an important natural antioxidant, may parallel the levels of serum GGT due to their relationship with oxidative stress, as reported in the Chinese population, especially in Chinese females (Zhang et al., 2015; Ya et al., 2017). Large cohort studies completed in recent years have found that high uric acid levels may increase the risk of cognitive impairment, possibly because uric acid is more damaging to vascular tissue than it is productive of antioxidant effects (Latourte et al., 2018; Singh and Cleveland, 2018). Therefore, the synergistic effect of GGT and SUA in MCI may be due to their effect on the risk of cardiovascular disease. In addition, a previous study found that increased SUA in women increases the risk of non-alcoholic fatty liver disease and that fatty liver disease development leads to increased GGT secretion, which might be an important reason for the parallel relationship between GGT and SUA levels (Chen et al., 2019).

In animal study, GGT levels have been found to increase significantly after ovariectomy, suggesting that estrogen levels have a significant effect on GGT expression (Zarida et al., 1994). At the same time, studies have shown that endogenous estrogens are associated with better cognitive performance, and our study found that a decrease in estrogens and an increase in GGT both affect MCI, this is of positive significance for the study of cognitive impairment in postmenopausal women (Fink et al., 2018; Shimizu et al., 2019).

In our study, we found that MCI is more common in women with elevated GGT levels, so GGT could be a potential diagnostic marker for MCI. Meanwhile, our findings indicated that women with high GGT levels had an increased risk of MCI when they were overweight, sleep deprived, or had high uric acid levels.

## Limitations

Since we performed a cross-sectional study, we could not refute the conclusion reached in previous studies that there is no causal link between GGT and MCI (Kunutsor et al., 2018). However, there has been no previous research into the relationship between GGT and cognitive function in the female population, so our results are significant in terms of the diagnosis and early indication of MCI in the female population. In addition, because our study only included Chinese women, the results may not be generalizable to the entire population. However, at the same time, our study was a two-center study that integrated analysis of different populations, and the conclusions are more representative and extrapolatable in Chinese female population.

## Data Availability Statement

The raw data supporting the conclusions of this article will be made available by the authors, without undue reservation.

## Ethics Statement

The study was conducted in accordance with the guiding principles of the Helsinki Declaration and approved by the Ethics Committee of Oilfield Inc. Medical Centers and Taian Traffic Hospital. Written informed consent was obtained from all participants.

## Author Contributions

All authors listed have made a substantial, direct and intellectual contribution to the work, and approved it for publication.

## Conflict of Interest

The authors declare that the research was conducted in the absence of any commercial or financial relationships that could be construed as a potential conflict of interest.
