# Supplementary figures and images for: Association Between Gamma-Glutamyl Transferase and Mild Cognitive Impairment in Chinese Women
Source: Front Aging Neurosci. 2021 Feb 10;13:630409. doi: 10.3389/fnagi.2021.630409 (PMC7902766; doi:10.3389/fnagi.2021.630409)

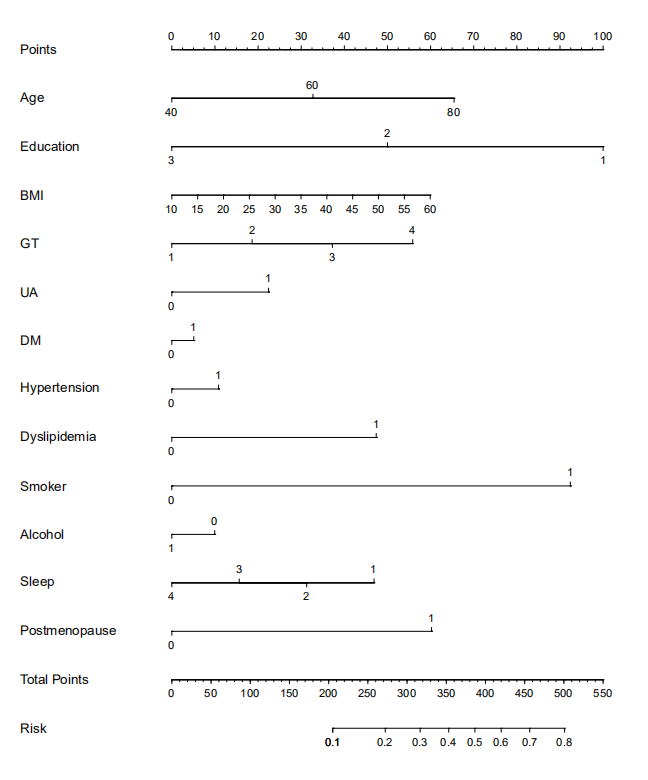

Supplement: Supplementary file 1 [file Image_1.tif]
