# Supplementary material for: Association Between Gamma-Glutamyl Transferase and Mild Cognitive Impairment in Chinese Women
Source: Front Aging Neurosci. 2021 Feb 10;13:630409. doi: 10.3389/fnagi.2021.630409 (PMC7902766; doi:10.3389/fnagi.2021.630409)
Supplement: Supplementary file 2 [file Table_1.docx]

**Table S1.** Differences in the baseline characteristics of participants in Jidong and Taian.

| Characteristic | Jidong | Taian |  |
| --- | --- | --- | --- |
|  | (n=2821) | (n=122) | P |
| GGT（U/L） | 20.9 (20.1) | 24.2 (21.3) | 0.109 |
| Age (years) | 45.8 ± 13.2 | 56.7 ± 8.6 | ＜0.001 |
| Age of MCI (years) | 59.8 (9.6) | 58.0 (4.7) | 0.204 |
| Education level (n, %) |  |  | 0.063 |
| <6 years | 167 (5.9) | 8(6.5) |  |
| 6-12 years | 943 (33.4) | 11(9.0) |  |
| >12 years | 1711 (60.6) | 103(84.4) |  |
| Sleep duration (n, %) |  |  | 0.080 |
| <7 hours | 1200 (42.5) | 60(49.1) |  |
| ≥7 hours | 1621 (57.4) | 62(50.8) |  |
| BMI (n, % ) |  |  | 0.053 |
| < 25 kg/m^2^ | 2021 (71.6) | 77(63.1) |  |
| ≥ 25 kg/m^2^ | 800 (28.4) | 45(36.9) |  |
| Current smoker (n, %) | 144 (5.1) | 7 (5.4) | 0.602 |
| Mild-moderate drinking (n, %) | 25 (0.8) | 3 (2.4) | 0.086 |
| Hypertension (n, %) | 296 (10.5) | 13 (10.6) | 0.841 |
| Dyslipidemia (n, %) | 164 (5.8) | 8 (6.5) | 0.439 |
| Diabetes (n, %) | 114 (4.0) | 8 (6.5) | 0.159 |
| UA（umol/L） | 297.1 ± 69.2 | 304.4 ± 67.8 | 0.071 |
| TC (mmol/L) | 5.4 ± 1.0 | 5.2 ± 0.9 | 0.635 |
| Triglycerides (mmol/L) | 1.5 ± 1.1 | 1.6 ± 1.0 | 0.092 |
| HDL‐C (mmol/L) | 1.3 ± 0.2 | 1.4 ± 0.3 | 0.426 |
| LDL‐C (mmol/L) | 2.1 ± 0.7 | 2.0 ± 0.6 | 0.895 |
| Menopause (n, %) | 949(33.6) | 43(35.2) | 0.713 |
