# Supplementary material for: Association Between Gamma-Glutamyl Transferase and Mild Cognitive Impairment in Chinese Women
Source: Front Aging Neurosci. 2021 Feb 10;13:630409. doi: 10.3389/fnagi.2021.630409 (PMC7902766; doi:10.3389/fnagi.2021.630409)
Supplement: Supplementary file 3 [file Table_2.docx]

**Table S2.** Differences in the baseline characteristics of participants with and without MCI.

| Characteristic | Total (n=2943) | MCI (n=444) | Non-MCI (n=2499) | *P* |
| --- | --- | --- | --- | --- |
| GGT (U/L) | 21.1 (20.9) | 24.6 (24.5) | 20.5 (20.1) | ＜0.001 |
| Age (years) | 46.3 ± 13.2 | 59.8 ± 9.5 | 43.9 ± 12.4 | ＜0.001 |
| Education level (n, %) |  |  |  | ＜0.001 |
| <6 years | 178(6.0) | 105 (23.6) | 73 (2.9) |  |
| 6-12 years | 1046(35.5) | 260 (58.6) | 786 (31.5) |  |
| >12 years | 1719(58.4) | 79 (17.8) | 1640 (65.6) |  |
| Sleep duration (n, %) |  |  |  | ＜0.001 |
| <7 hours | 1262(42.9) | 272 (61.3) | 990 (39.6) |  |
| ≥7 hours | 1681(57.1) | 172 (38.7) | 1509 (60.4) |  |
| BMI (n, % ) |  |  |  | ＜0.001 |
| < 25 kg/m^2^ | 2098(71.3) | 252 (56.8) | 1846 (73.9) |  |
| ≥ 25 kg/m^2^ | 845(28.7) | 192 (43.2) | 653 (26.1) |  |
| Current smoker (n, %) | 144(4.9) | 10 (2.3) | 134 (5.3) | 0.001 |
| Mild-moderate drinking (n, %) | 28(1.0) | 4 (0.9) | 24 (1.0) | 0.905 |
| Hypertension (n, %) | 319(10.8) | 114 (25.7) | 195 (7.8) | ＜0.001 |
| Dyslipidemia (n, %) | 172(5.8) | 68 (15.3) | 104 (4.2) | ＜0.001 |
| Diabetes (n, %) | 122(4.1) | 46 (10.4) | 76 (3.0) | ＜0.001 |
| UA (umol/L) | 297.8 ± 69.3 | 312.8 ± 73.4 | 295.1 ± 68.2 | ＜0.001 |
| TC (mmol/L) | 5.6 ± 7.0 | 5.6 ± 3.8 | 5.6 ± 7.4 | 0.994 |
| Triglycerides (mmol/L) | 1.5 ± 1.1 | 1.9 ± 1.4 | 1.4 ± 1.0 | ＜0.001 |
| HDL‐C (mmol/L) | 1.3 ± 0.3 | 1.3 ± 0.2 | 1.3 ± 0.3 | 0.900 |
| LDL‐C (mmol/L) | 2.0 ± 0.7 | 2.3 ± 0.7 | 1.9 ± 0.7 | ＜0.001 |
| Menopause (n, %) | 992(33.7) | 293(65.9) | 699(27.9) | ＜0.001 |
