# Supplementary material for: Association Between Gamma-Glutamyl Transferase and Mild Cognitive Impairment in Chinese Women
Source: Front Aging Neurosci. 2021 Feb 10;13:630409. doi: 10.3389/fnagi.2021.630409 (PMC7902766; doi:10.3389/fnagi.2021.630409)
Supplement: Supplementary file 4 [file Table_3.docx]

**Table S3.** The effects of high GGT levels, sleep deprivation, hyperuricemia and overweight on MCI

| Characteristic | Model1 | Model2 | Model3 |
| --- | --- | --- | --- |
| High GGT | 1.29 (1.17,1.42) | 1.21 (1.09,1.35) | 1.19 (1.07,1.33) |
| Overweight | 1.84 (1.48,2.28) | 1.34 (1.05,1.71) | 1.32 (1.04,1.69) |
| Insufficient sleep | 2.29 (1.86,2.83) | 1.38 (1.09,1.74) | 1.33 (1.05,1.69) |
| Hyperuricemia | 1.77 (1.39,2.26) | 1.39 (1.06,1.83) | 1.34 (1.01,1.77) |
| Menopause | 4.99(4.03,6.19) | 2.39(1.88,3.06) | 2.28(1.78,2.91) |

Model 1: unadjusted

Model 2: adjusted for age, education, smoking status, and alcohol consumption.

Model 3: adjusted for age, education, smoking status, alcohol consumption, hypertension, hyperlipidemia, and diabetes mellitus.

High GGT, GGT level≥quartile 4; Overweight, BMI≥25 kg/m^2^; Insufficient sleep, sleep duration<7 hours; Hyperuricemia, UA level≥360 umol/L
